# QTLs and eQTLs mapping related to citrandarins’ resistance to citrus gummosis disease

**DOI:** 10.1186/s12864-018-4888-2

**Published:** 2018-07-03

**Authors:** Rômulo P. M. Lima, Maiara Curtolo, Marcus V. Merfa, Mariângela Cristofani-Yaly, Marcos A. Machado

**Affiliations:** 1 Centro APTA Citros Sylvio Moreira, Centro de Citricultura Sylvio Moreira, Instituto Agronômico (IAC), CP 04, Cordeirópolis, SP 13490-970 Brazil; 2 Departamento de Genética, Instituto de Biociências, UNESP, Caixa Postal 510, CEP, Botucatu, SP 18618-000 Brazil; 3 Department of Entomology and Plant Pathology, Auburn University, Auburn, AL 36849 USA

**Keywords:** *Citrus sunki*, Defense genes, Hybrids, Expression quantitative trait loci, *Poncirus trifoliata*

## Abstract

**Background:**

*Phytophthora nicotianae* Breda de Haan (*Phytophthora parasitica* Dastur) causes severe damage to citrus crops worldwide. A population of citrandarins was created from the cross between the susceptible parent *Citrus sunki* Hort. Ex Tan. and the resistant parent *Poncirus trifoliata* (L.) Raf. cv. Rubidoux, both parents and two reference rootstocks (Rangpur lime and Swingle citrumelo) were grafted in a greenhouse on Rangpur lime. Inoculations were performed at 10 cm and 15 cm above the grafting region and the resulting lesions were evaluated by measuring the lesion length 60 days after inoculation. As control, non-inoculated plants of each genotype were used. In addition, we evaluated the expression of 19 candidate genes involved in citrus defense response 48 h after pathogen infection by quantitative Real-Time PCR (qPCR). We mapped genomic regions of Quantitative Trait Loci (QTLs) and Expression Quantitative Trait Loci (eQTLs) associated with resistance to *P. parasitica* in the linkage groups (LGs) of the previously constructed maps of *C. sunki* and *P. trifoliata*.

**Results:**

We found disease severity differences among the generated hybrids, with lesion lengths varying from 1.15 to 11.13 mm. The heritability of the character was 65%. These results indicate that there is a great possibility of success in the selection of resistant hybrids within this experiment. The analysis of gene expression profile demonstrated a great variation of responses regarding the activation of plant defense pathways, indicating that citrandarins have several defense strategies to control oomycete infection. The information of the phenotypic and gene expression data made possible to detect genomic regions associated with resistance. Three QTLs and 84 eQTLs were detected in the linkage map of *P. trifoliata*, while one QTL and 110 eQTLs were detected in *C. sunki*.

**Conclusions:**

This is the first study to use eQTLs mapping in the *Phytophthora*-citrus interaction*.* Our results from the QTLs and eQTLs mapping allow us to conclude that the resistance of some citrandarins to the infection by *P. parasitica* is due to a favorable combination of QTLs and eQTLs transmitted by both parents.

**Electronic supplementary material:**

The online version of this article (10.1186/s12864-018-4888-2) contains supplementary material, which is available to authorized users.

## Background

Hybrids of microtangerines, such as the ones obtained from the cross of Sunki mandarin (*Citrus sunki* Hort. Ex Tan.) with trifoliate [*Poncirus trifoliata* (L.) Raf.], are called citrandarins. They are part of a new generation of rootstocks for citrus and combine the advantageous characteristics of mandarins, such as tolerance to Citrus blight and Citrus sudden death, with the qualities of trifoliates, which correspond to high resistance to *Phytophthora*, immunity to *Citrus tristeza virus* and resistance to citrus nematode, besides the great ability to form compact, productive plants with good fruit quality [1]. In a study on the *Phytophthora*-Citrus pathosystem, a F1 population from the crossing between *C. sunki* (susceptible parent) and *P. trifoliata* cv. Rubidoux (resistant parent) was obtained and evaluated for *Phytophthora* gummosis. Differences in the level of symptoms in both parents and progeny were observed [2].

Root rot, caused by *Phytophthora*, and gummosis and its various synonyms (foot rot, neck rot and trunk gum disease) are among the diseases that damage citrus crops. Citrus nurseries and orchards worldwide have been severely damaged by *Phytophthora nicotianae* Breda de Haan (*Phytophthora parasitica* Dastur) and *Phytophthora citrophthora* (Smith & Smith). In Brazil, *P. parasitica* is the most predominant species associated with the disease [3]. These pathogens can infect the main branches of the plants, inducing the formation of cankers with gum exudation of the lesions [3, 4]. Infected plants usually lose their vigor and may die prematurely [5].

*P. parasitica* is an oomycete, which belongs to the kingdom Stramenopila, comprising a diversified group of organisms [6]. During infection, oomycetes establish intimate relationships with their hosts through the formation of haustoria, structures used to obtain nutrients from plants, reprogramming the host defense metabolism. Species from the genus *Phytophthora* are hemibiotrophic pathogens, which means they initiate the infection as biotrophic agents in the first 36–48 h and colonize the host as necrotrophic pathogens, alternating the way of obtaining nutrients in order to guarantee its dissemination from necrotic plant tissues [7]. Gummosis is considered the most serious disease caused by *Phytophthora* spp. The lesions caused by the *Phytophthora* grow around the tree trunk damaging the cambium and inner bark killing it [8].

It is known that more than one mechanism of resistance and/or tolerance are involved in the citrus defense response to *Phytophthora* due to the large differences in types of affected tissues, and consequently to different citrus species’ responses to the infection. In general, responses that are common to all plant species affected by phytopathogens can be activated, such as hypersensitivity reaction (HR), the development of structural barriers and the production of antimicrobial compounds called phytoalexins. In addition, induction of specific responses to a given population of *Phytophthora* may occur, which are controlled by host resistance genes [9].

The great volume of information generated by genome sequencing projects has allowed a large-scale approach to differential gene expression analysis using techniques such as microarray, RNA Sequencing and qPCR (Real Time Quantitative PCR). Some information has already been produced to elucidate the molecular basis of the host response to *Phytophthora* gummosis disease [10, 11]. These studies have reported on changes in global gene expression profile and have shown differentially expressed genes involved in processes such as cellular defense and metabolism of carbohydrates, lipids and proteins. Defense genes are activated in response to pathogen infection by signal transduction mechanisms, which are regulated by phytohormones pathways, with salicylic acid (SA), jasmonic acid (JA), ethylene (ET) and abscisic acid (ABA) being the most common ones [12].

According to Pieterse et al. [13], the crosstalk that occurs between the SA, JA and ET pathways confers a directed defense response against different plant pathogens. There is a synergy between the JA and ET pathways, while there is a mutual repression between these two pathways with the SA pathway. In addition, it has been shown that ABA, which is known as the abiotic stress hormone in plants and is considered a negative regulator of resistance to phytopathogens, may be involved in increasing plant resistance to disease through its positive effect on callose deposition. ABA may also contribute to plant defense by the convergence of signaling with the JA pathway, which synergistically triggers the expression of defense genes responsive to both hormones [13, 14].

Genomics emerges as a technique that encompasses quantitative loci mapping and gene expression analysis to identify the association between the allelic status of a genome region and the quantification of gene transcripts [15]. These genomic regions are referred to as Expression Quantitative Trait Loci (eQTLs). The study of QTLs and eQTLs is of great importance for understanding pathogen-host interaction and to understand mechanisms of resistance and response to diseases.

In this study, we mapped the genomic regions associated with resistance to *P. parasitica* by means of phenotypic (QTLs) and expression (eQTLs) analyses in the linkage groups (LGs) of previously constructed maps of *C. sunki* and *P. trifoliata*. We evaluated the resistance of *C. sunki* x *P. trifoliata* hybrids to *P. parasitica*, as well as the expression of candidate genes related to resistance to *P. parasitica* by qPCR in the parents and hybrids used in the experiment.

## Methods

### Plant material and inoculation of *P. parasitica*

A population of 110 hybrids of *C. sunki* (susceptible to *Phytophthora*) x *P. trifoliata* (resistant) and parents was established in a greenhouse. Rangpur lime (*Citrus limonia* Osbeck) and Swingle citrumelo [*Citrus paradisi* Macfad. cv. Duncan x *Poncirus trifoliata* (L.) Raf.], two reference rootstocks in citrus cultivation, were also evaluated. Plants were multiplied by bud grafting using Rangpur lime as rootstock, and were kept in a greenhouse until the stem reached a diameter of approximately 0.8 to 1.0 cm.

The isolate IAC-01/95 from the isolates’ collection of the Plant Pathology Clinic of Sylvio Moreira Citrus Center, which was obtained from infected soil, was used in this assay. Mycelial disks of the pathogen growing in carrot-agar culture medium [16], containing 1 ml of Rifampicin, 1 ml of Ampicillin and 2 ml of Benomyl per 1000 ml of the medium, were removed from the colonies and transferred to Petri dishes containing 15 ml of this medium.

To activate the pathogenicity of the isolate, a re-isolation was performed from the lesion sections of tissue infected with the pathogen’s mycelium (pre-inoculation). Small portions of the lesions were taken and inoculated in Sicilian lemon fruits. These fruits were incubated at 25 °C with a photoperiod of 16 h for nine days. Then, they were carefully opened under brief asepsis inside a flow cabinet. With the disease affecting a large area of the fruit, the contaminated seeds were removed and transferred to Petri dishes containing the selective culture medium with carrot-agar. They were subsequently kept at 25 °C in the dark for six days, until the formation of the pathogenic mycelium.

The experimental design was a completely randomized assay with two replicates of each genotype and one plant per plot. Each hybrid was conducted with a single stem and inoculation was performed at 10 cm and 15 cm above the grafting region, totalizing two inoculations per genotype, as described by Siviero et al. [2]. This method consists of: disinfestation with alcohol of the surface of the trunk region to be inoculated; incision in the bark using a cork borer and removing a 5 mm diameter disc, thus exposing the cambium zone; introduction of a disk of equal diameter of virulent *P. parasitica* mycelium maintained in culture medium; placing back the same disc of the bark and protection of the wound site caused by inoculation with cotton and tape. As a control, and to remove the effect of the stress caused by the wound, only the incision itself was made in the bark of plants with a cork borer, without inoculation of the pathogen, in each genotype of the experimental population.

### Evaluation of lesions and statistical analysis of phenotypic data

To evaluate the lesions, plants were maintained in an artificial lighting environment with a photoperiod of 16 h, a temperature of 25 °C and a relative humidity of 85%, with daily irrigations. The resulting lesions were evaluated 60 days after inoculation by measuring the length of the injured area after bark removal of the trunk with the aid of a digital caliper, totally exposing the developed lesion.

For the normality’s hypothesis of the data and for the statistical analysis, were used the average lengths of the lesions from inoculated individuals (LLI - Lesion length by inoculation) minus the average lengths obtained from control individuals (LLC – Lesion length in control), which were around 5 mm, due to the cork borer’s diameter used to cause injury to the plant, resulting in the variable RLL (Real lesion length) [LLI - LLC = RLL]. A QQ-plot for the distribution of lesion means was obtained using the Minitab 18 software (http://www.minitab.com/), based on the Kolmogorov-Smirnov normality test, with log (base 10)-transformed phenotypic data.

Statistical analyses were performed using the SASM-AGRI software [17], applying the F-test for analysis of variance and using the appropriate statistical model in a completely randomized design. In addition, the Scott-Knott test for comparison of treatments means was also used.

The averages of the phenotypic data from the RLL evaluation of the progeny of the crossing between *P. trifoliata* and *C. sunki* were used to calculate the genetic parameters (heritability, variance, and coefficient of variation) for the variable measured using the model 83 of the SELEGEN-REML/BLUP software [18].

### Leaf collection, RNA extraction and cDNA synthesis

Leaf collection was performed 48 h after inoculation of the pathogen in the plants. Leaves were placed in liquid nitrogen and stored in a − 80 °C freezer until the extraction of RNA. The choice of collection time was based on previous time course studies that evaluated the systemic response of plants using the expression of genes involved in the *Phytophthora*-citrus interaction [11, 19] and associated with the signaling pathways of phytohormones that activate several defense mechanisms in the plant.

Total RNA extraction from plant material was performed according to the lithium chloride (LiCl) method described by Chang et al. [20]. To avoid contamination with genomic DNA, samples were treated with DNase I, RNase-Free kit (Fermentas Life Sciences), following the manufacturer’s recommendations. 300 mg of leaves from the same genotype were ground with liquid nitrogen and three tubes of the resulting macerate (100 mg each) were collected, totaling three biological replicates per genotype. Total RNA was then quantified in NanoDrop ND-8000 spectrophotometer (Thermo Scientific), and its integrity was evaluated with 1.2% denaturing agarose gel electrophoresis.

The cDNA was synthesized according to instructions from the Revertaid H Minus First Strand cDNA Synthesis Kit (Fermentas), using oligo (dT) primers, and then treated with RNAse H (1 U) to remove any RNA contamination. Subsequently, the obtained cDNA was diluted in RNAse-free water at the ratio of 1:50, with the three biological replicates of cDNA (50 μL each) being mixed. This formed a pool of samples represented in a single tube containing 150 μL of diluted cDNA from each genotype to be analyzed in gene expression and eQTL mapping assays.

### qPCR for validation of gene expression

The expression of 19 candidate genes by qPCR (Additional file 1: Table S1), selected in previous studies with microarray and gene expression [10, 11], was evaluated in 51 hybrids, the two parents (*C. sunki* and *P. trifoliata*), Rangpur lime and Swingle citrumelo. The selection of hybrids for the qPCR assays was performed based in the application of the Scott-Knott test on the phenotypic data, which were separated into three groups (susceptible [S]; moderately susceptible [MS]; and tolerant and/or resistant [T/R]). Six hybrids were selected and formed the S group together with the two susceptible rootstocks (*C. sunki* and Rangpur lime). Three hybrids formed the MS group, and 42 hybrids together with the two resistant rootstocks (*P. trifoliata* and Swingle citrumelo) formed the T/R group. In general, we screened for genes related to cellular defense processes, which involve the main pathways of phytohormones such as SA, JA, ET and ABA. Moreover, some genes, such as *LEA5* and *RPS4*, found differentially expressed by microarray among *P. trifoliata*, *C. sunki* and in some hybrids, in order to be a strong candidate in *Phytophthora* resistance [10], and other genes, such as *PR2*, *PR3* and *PAL*, which were induced within 48 h after infestation in previous time course studies evaluating target genes related to the pathosystem formed by *Phytophthora* with two citrus species phenotypically opposed to the invader, corresponding to *P. trifoliata* (resistant) and *C. sunki* (susceptible) [11], were also evaluated. As endogenous controls, the *FBOX* (F-box family protein), *SAND* (SAND protein family) and *GAPC2* (Glyceraldehyde-3-phosphate dehydrigenase C2) genes were analyzed.

The oligonucleotides primers flanking the gene regions were synthesized using the Primer Express 2.0 software (Applied Biosystems), and checked for specificity by Primer-BLAST tool in the NCBI (National Center for Biotechnology Information) database. Their sequences were found in the literature, in studies evaluating qPCR experiments with Arabidopsis, tobacco, rice and in the CitEST database for citrus plants.

The amplifications by qPCR for the gene expression assays were performed using 7.5 μL of GoTaq qPCR Master Mix (Promega), 3 μL of cDNA (1:50 dilution), which was taken from the pool formed by the three replicates (150 μL each), 200 nM of each primer and water to complete a final volume of 15 μL, and carried out in a 7500 Fast Real Time PCR System (Applied Biosystems) with cycles of: 50 °C for 2 min; 95 °C for 10 min; 40 cycles of 95 °C for 15 s and 60 °C for 1 min. For each reaction, a dissociation curve was performed to verify possible non-specific contaminations and reactions. In addition, the reactions for each pair of primers were made in duplicates, always using control without cDNA to detect possible contaminations.

### Normalization of qPCR data and gene expression analyses

Expression stability for each housekeeping gene was evaluated using the GenEx v. 6 (http://www.multid.se/) software, which provides the GeNorm algorithm to make paired comparisons between the endogenous genes, determining the best combination (two or more genes) to be used and calculating the average value of the Expression stability, called the M value (m-value) for each gene. The data that served as input for the calculation of the M value by the GeNorm algorithm were the relative quantities of each sample together with the efficiency of the primer, according to Mafra et al. [21].

For the detection of relative quantities, expression levels, fold change values ​​and stability of endogenous genes, two treatments were considered in the study of the *Phytophthora*-citrus complex: plants inoculated with *P. parasitica* (infected group) and non-inoculated plants (control group). Calculations were made based on the number of amplification cycles required to reach a fixed threshold (cut-off cycle - Ct) in the exponential phase of PCR. The amplification efficiency and Ct data were calculated for each qPCR reaction by the Real-time PCR Miner software [22], which implements a normalizing algorithm to the raw fluorescence data according to the function of the PCR cycles. For each gene, an average efficiency of all PCRs was calculated. The mean of the Ct values ​​of the two technical replicates of each genotype analyzed was also estimated (Additional file 2: Table S2). With these data, the relative quantification was calculated. It was determined by the 2^-ΔΔCT^ method between the conditions of qPCR [23], which serves as parameter for the calculation of the expression levels of each sample. Hence, a methodology derived from this procedure was established, which does not consider the efficiency value (E) of the gene equal to 100%. Instead, the actual value of E was used in the calculations of the relative quantities (Q) of each sample by the formula Q = E^ΔC T^.

Expression levels of each genotype were calculated by a normalization factor, given by the geometric mean of the estimated relative quantities for the two reference genes that had a smaller M value in the two treatments evaluated by the GeNorm algorithm. The calculation of expression levels in each sample was done by the ratio between the value of the relative quantity found in the target gene and the normalization factor of that genotype under analysis.

At last, the Fold change values ​​of each inoculated individual were calculated in relation to the non-inoculated ones, by the ratio of the expression levels of the infected group on the control group. For statistical analysis, Fold change data were transformed into logarithmic scale (base 2), to meet the data normality using the Minitab 18 software (http://www.minitab.com/). An F-test (ANOVA: a criterion) was made to compare log_2_Fold change results for each gene between the three groups (S, MS and T/R). If the obtained *p*-value was significant, a *post-hoc* paired Student’s t-test was applied to compare the differences between the sample means of the three groups.

### Gene expression profile

To obtain the gene expression profile of the experiment, by comparing the 19 candidate genes among the 51 hybrids, the two parents, Rangpur lime and Swingle citrumelo, Log_2_Fold change values ​​served as input to the MeV (MultiExperiment Viewer) program v. 4.9 (http://sourceforge.net/projects/mev-tm4/). It was possible to group both the genes and the samples by the Hierarchical clustering (HCL), using the Pearson correlation as metric distance, which resulted in the gathering of genes with closer expressions and the separation of the genotypes in clusters. These parameters were visualized in a graphical representation called heatmap.

In addition, to better understand the relationship between the candidate genes, a dissimilarity matrix was obtained from the Euclidean Distance, so that this comparative analysis could also be demonstrated with a heatmap.

### QTLs and eQTLs mapping

In the present study, we used pre-built linkage maps of *C. sunki* and *P. trifoliata* [24], that were constructed using DArT-seq™ molecular markers and the OneMap software [25]. The analyses were performed using the FullsibQTL package of the programming language R [26]. For the QTLs mapping, the means of the RLL phenotypic data in logarithmic transformation (base 10) in the segregating population of 110 hybrids were considered. For the eQTLs mapping, the Log_2_Fold change values ​​calculated by the relative expression in the segregating population composed of 51 selected hybrids were used to map the eQTLs on the two parental linkage maps, as well as the phenotypic trait, thus detecting polymorphisms associated with gene expression. The strategy of composite interval mapping (CIM) [27] was used. A maximum of 20 cofactors were stipulated to locate QTLs and eQTLs outside the mapping range. The calculation of the critical LOD score to determine the presence of true QTL and eQTL was done by the random permutation test (α = 0.05; *n* = 1000 replicates) [28], available in FullsibQTL. The percentages of phenotypic variation (R^2^) explained by QTLs and eQTLs were also estimated.

## Results

### Evaluation of lesions caused by *P. parasitica* and phenotypic analyses

**Fig. 1:**
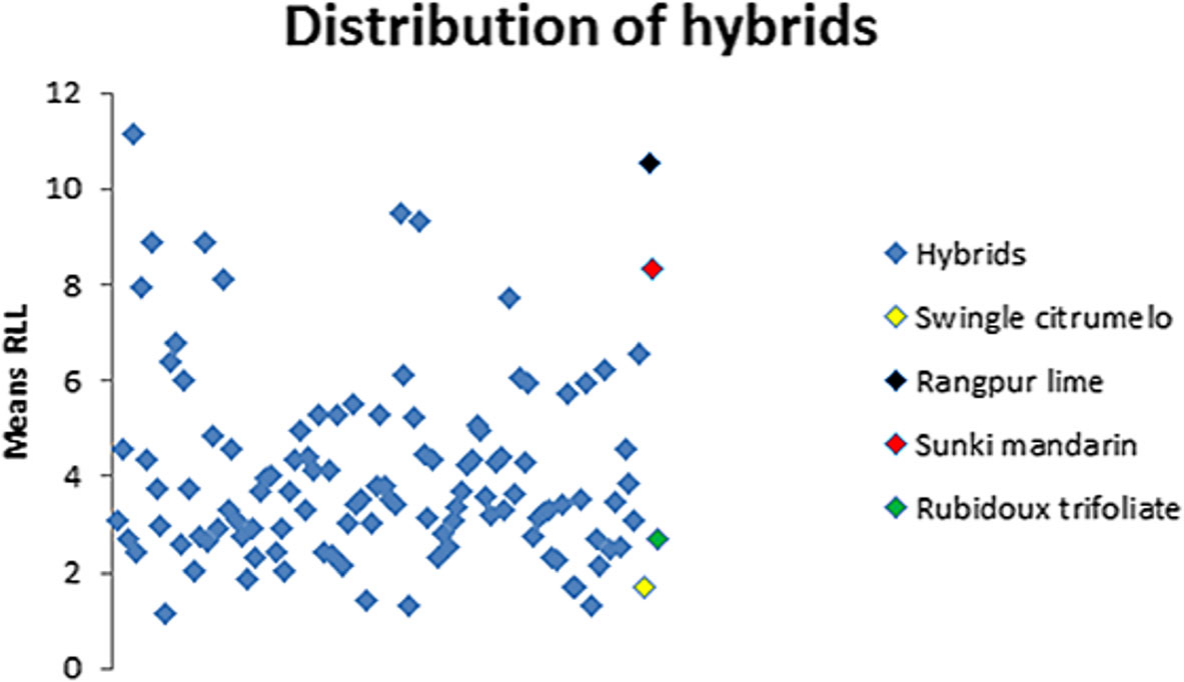
Means’ distribution of real lesion length (RLL) in the individuals of experiment

The means of lesions by inoculation of the 110 hybrids, *C. sunki*, *P. trifoliata*, Rangpur lime and Swingle citrumelo with *P. parasitica* are shown in Fig. 1.

The RLL from the population composed by *P. trifoliata*, *C. sunki*, the 110 hybrids, Rangpur lime and Swingle citrumelo met the expectation of normal distribution data by the Kolmogorov-Smirnov (KS) normality test.

There were significant differences between the RLL means of the 114 genotypes (Additional file 3: Table S3). It can be stated that the genotypes Rangpur lime, Sunki mandarin, hybrids 8, 204, 209, 40, 13, 847, 11 and 244 are susceptible to *P. parasitica*, because they presented higher RLL values. Hybrids 30, 312, 27, 284, 205, 248, 33, 274, 250, 262 and 99 showed moderate susceptibility to the disease, with intermediate RLL values. On the other hand, the remaining 93 genotypes behaved as tolerant and/or resistant to *Phytophthora*, including *P. trifoliata* and Swingle citrumelo, because they presented smaller RLL values.

Broad-sense heritability coefficient was considered high (65%) for the RLL character (Table 1).

**Table 1:** Estimates of genetic parameters for Real lesion length (RLL) character

Estimates^a^	RLL (mm)
V_g_	2.8
V_r_	1.48
V_p_	4.28
H^2^	0.65 ± 0.15
CV_g_%	41.76
CV_r_%	30.35
Overall mean	4.0065

^a^genotipic variance (V_g_); residual variance (V_r_); phenotypic variance (V_p_); broad-sense heritability coefficient (H^2^); coefficient of variation for genotypic variance (CV_g_%); coefficient of variation for residual variance (CV_r_%) and overall character mean

### Gene expression and genotypic analyses

Three endogenous normalizing genes were analyzed and all of them presented high expression stability (Table 2), with M values varying from 0.16 for the best candidates to Normalizers (*FBOX* and *SAND*) at 0.21 (*GAPC2*), which had the highest m-value recorded. Therefore, the *FBOX* and *SAND* genes were selected as normalizing genes for the analyses of candidate genes, related to resistance to *P. parasitica*.

**Table 2:** Normalizing genes evaluated by the GeNorm algorithm, with the values of mean Ct, standard deviation (SD) of Ct values and stability M value (m-value)

Gene	Mean Ct^a^	SD^a^	m-value
FBOX	28.06	1.88	0.16
SAND	31.29	1.78	0.16
GAPC2	24.18	2.10	0.21

^a^Mean of Ct data and standard deviation of Ct data by qPCR reactions using duplicates of 55 samples related to two experimental treatments (inoculation and control)

The results of the relative gene expression in the population composed of *P. trifoliata*, *C. sunki*, 51 hybrids, Rangpur lime and the Swingle citrumelo satisfied the expectation of normal distribution. The *p*-value obtained by the KS test was bigger than 0.150 for 17 analyzed genes, which made the log_2_Fold change values ​​highly significant (*p* ≥ 0.01 and *p* ≥ 0.05) and allowed the development of the following comparative analyses. Only the *PR2* and *PR4* genes had a smaller *p*-value (0.038 and 0.077, respectively).

The F-test (ANOVA: a criterion) results for variance analysis using log_2_Fold change values ​​from 19 target genes evaluated in the *P. trifoliata*, *C. sunki*, 51 hybrids, Rangpur lime and Swingle citrumelo revealed that only five genes (*EDS1*, *WRKY46*, *WRKY62*, *Chitinase* and *LEA5*) had significant different expression among the three groups (S, MS and T/R). The *EDS1*, *WRKY46* and *LEA5* genes had a smaller *p*-value (0.0079, 0.0073 and 0.0022, respectively), and were highly significant at 1% (*p*-value ≤0.01) compared to *WRKY62* and *Chitinase* (0.0254 and 0.0495, respectively), with significant difference at 5% (*p*-value ≤0.05).

**Fig. 2:**
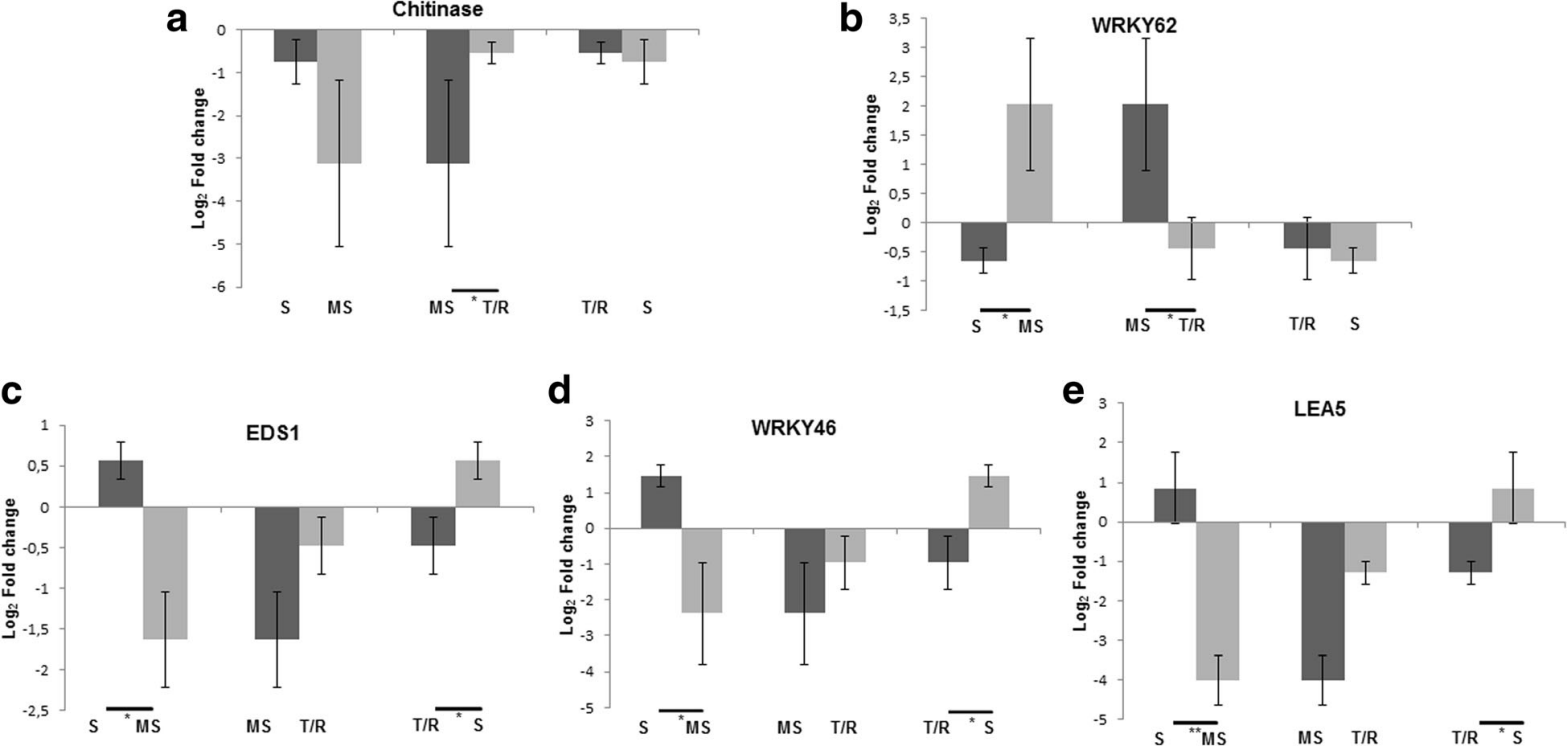
Paired Student’s t-test, showing the expression pattern per grouping in which there was a significant difference by F-test. S = susceptible; MS = moderately susceptible; T/R = tolerant and/or resistant. Five genes are shown: **a** = Chitinase, **b** = WRKY62, **c** = EDS1, **d** = WRKY46 and E = LEA5. The log_2_Fold change values are represented in relation to the non-inoculated control (log_2_Fold change = 0). *Difference (α = 0.05) between groups in pairs. **Difference (α = 0.01) between groups in pairs

After the F-test, a paired Student’s t-test was applied to the five genes with statistically significant difference, comparing the sample means of the three groups in pairs (Fig. 2).

**Fig. 3:**
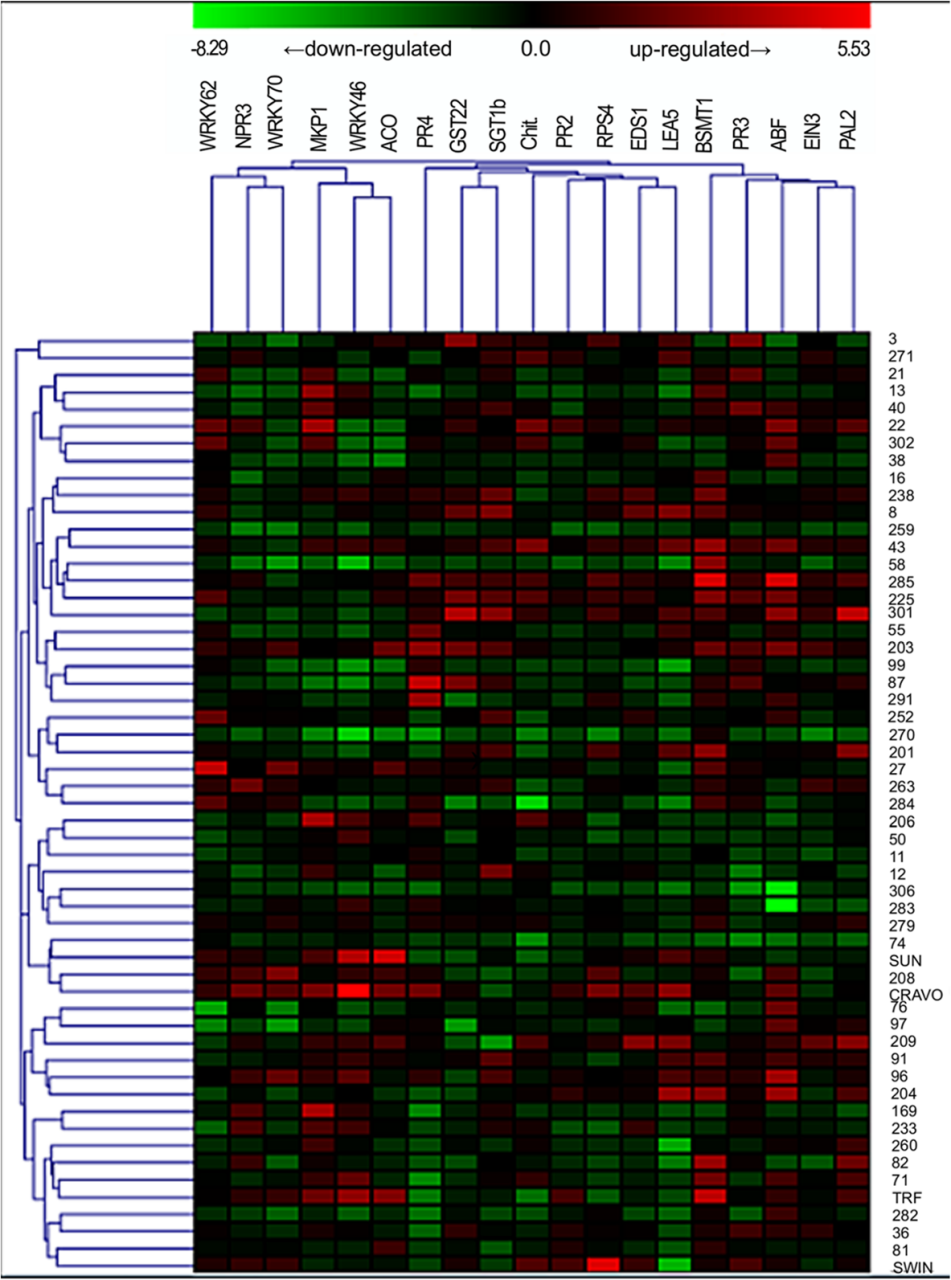
Heatmap of the gene expression profile by clustering analyses between 19 evaluated target genes with 55 selected genotypes (51 hybrids, Rubidoux trifoliate [TRF], Sunki mandarin [SUN], Rangpur lime [CRAVO] and Swingle citrumelo [SWIN]). The heatmap was made using Log_2_Fold change normalized data as input to the MeV (MultiExperiment Viewer) program v. 4.9 (http://sourceforge.net/projects/mev-tm4/). Names of genes and gene hierarchical cluster are shown in the top of the figure. Log_2_Fold change expression values representation ranges from red (highest expression) to green (lowest expression). Sample names (55 selected genotypes) are shown on the right side of the figure, while sample hierarchical cluster is shown on the left side

The heatmap (Fig. 3), based on the comparative analysis performed by the Hierarchical clustering (HCL) of 19 target genes and the 55 selected genotypes (51 hybrids, their two parents, Rangpur lime and Swingle citrumelo), allowed the grouping of genes and related genotypes, using the Pearson correlation as metric distance to obtain the best intra and intervariable grouping possible. The genotypes were separated into five secondary clusters distributed in two main clusters, while the genes in three secondary clusters arranged in two main clusters. The color scale representing the log_2_Fold change values showed that the gene expression data ranged from − 8.29 to 5.53.

**Fig. 4:**
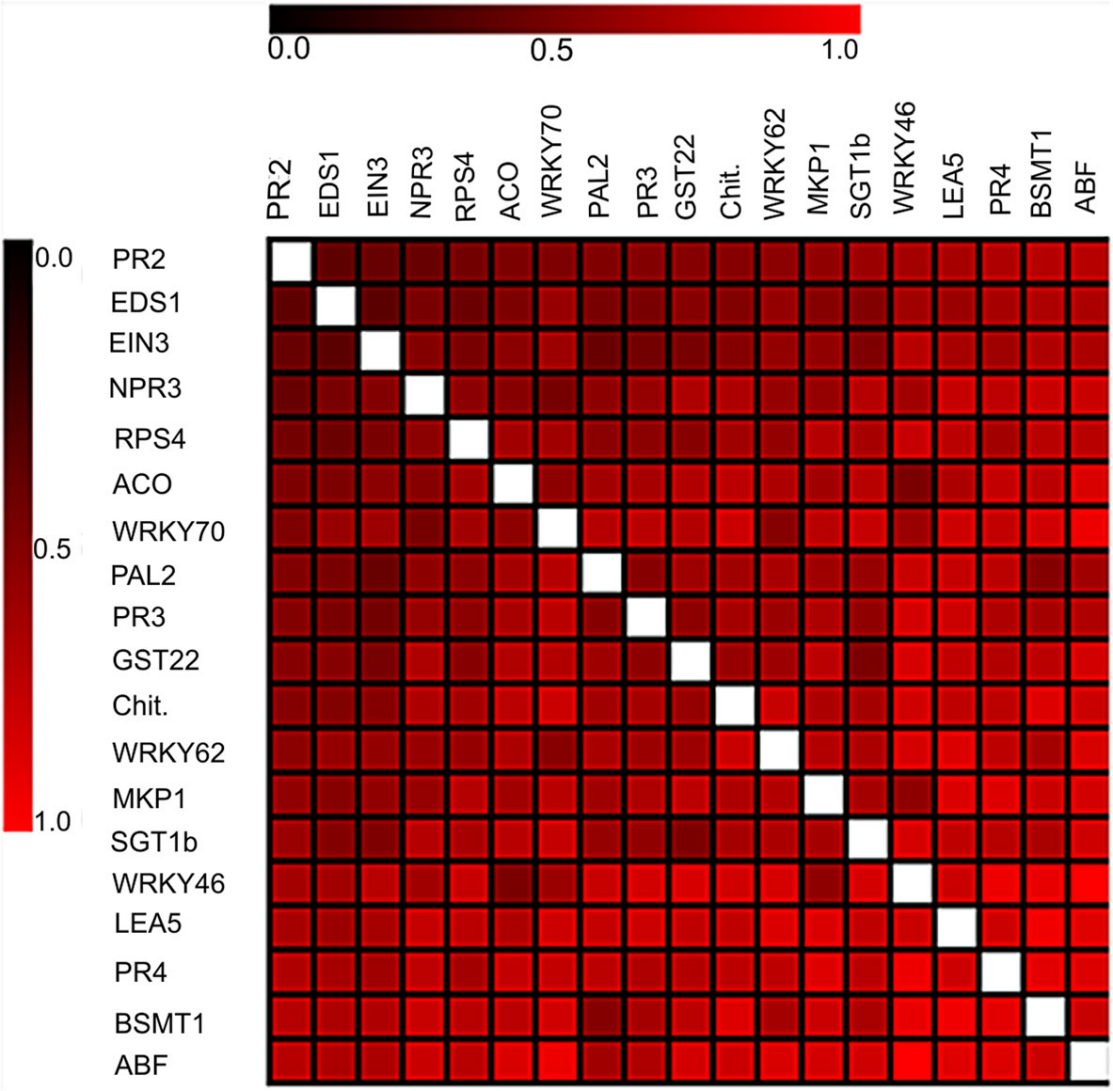
Heatmap representing the global dissimilarity matrix between 19 evaluated target genes from the paired comparison of genes using the Euclidean Distance. The heatmap was made using Log_2_Fold change normalized data as input to the MeV (MultiExperiment Viewer) program v. 4.9 (http://sourceforge.net/projects/mev-tm4/). Color shades in the dissimilarity matrix represent degree of similarity: dark red indicates high similarity, light red high dissimilarity

Another heatmap (Fig. 4) was constructed for a more accurate analysis of the relationship among the candidate genes, from a dissimilarity matrix, which uses as a metric distance the measure called Euclidean Distance, allowing to observe the difference degree among genes in a range of 0 to 1.

### QTLs analysis and mapping

Using the two genetic maps previously constructed for each parent of the hybrids progeny [22] and according to RLL phenotypic data, it was possible to detect QTLs associated with the lesion length character caused by *Phytophthora* in citrus.

**Fig. 5:**
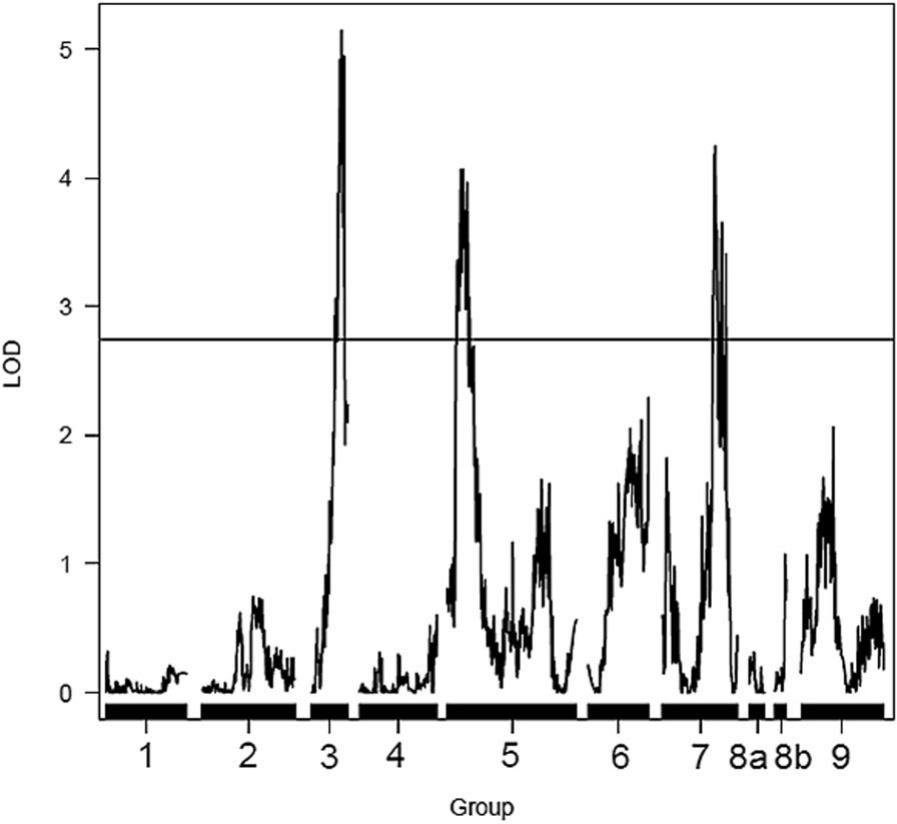
Detection of QTLs related with the resistance to *Phytophthora parasitica* in the *Poncirus trifoliata* linkage map. Y-axis: LOD; X-axis: distance in centiMorgans; LOD score = 2.74

**Fig. 6:**
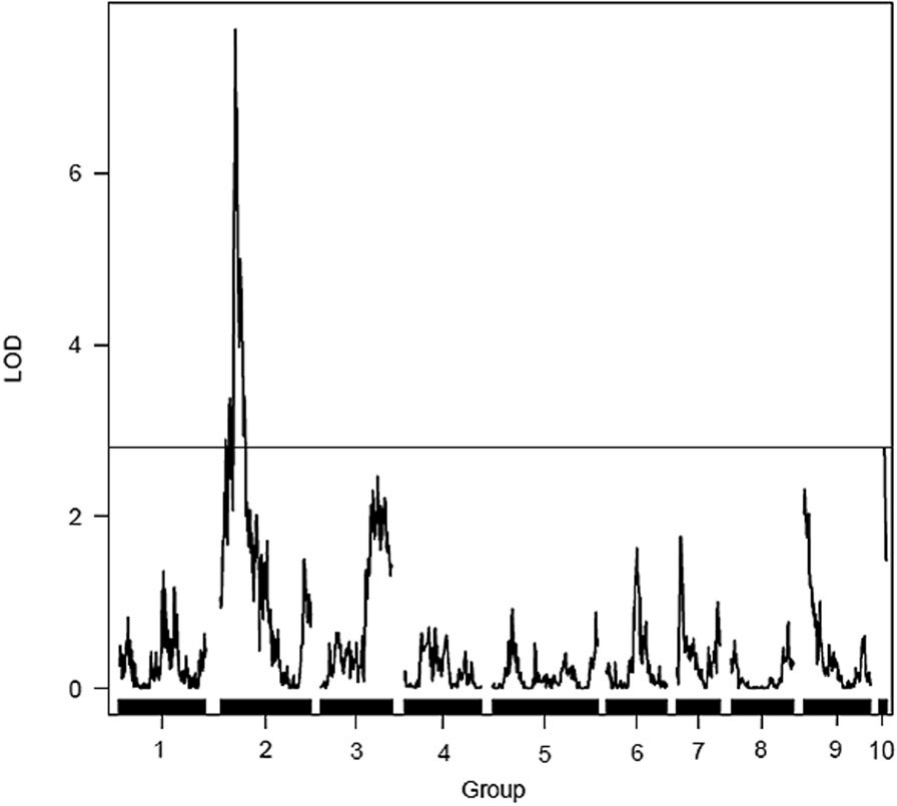
Detection of one QTL related with the resistance to *Phytophthora parasitica* in the *Citrus sunki* linkage map. Y-axis: LOD; X-axis: distance in centiMorgans; LOD score = 2.81

Based on the methodology of composite interval mapping (CIM), four QTLs were detected with LOD score higher than or equal to 3.0, three for the *P. trifoliata* (resistant parent) map and one for the *C. sunki* (susceptible parent) map (Figs. 5 and 6). In this model, only the additive effect was estimated, and the signal indicates the linkage phase between the marker and the QTL.

QTLs were found in LGs 3, 5 and 7 of the *P. trifoliata* map with 1:1 segregation due to the significant additive effect for this parent (Fig. 5). Regarding the QTLs analysis in the *C. sunki* linkage map, there was only one QTL located in LG 2 that showed 1:1 segregation ratio, also with significant additive effect for this parent (Fig. 6).

Table 3 shows the results of the QTLs mapping. “LOD scores” of the detected QTLs ranged from 4.06 to 7.67 while R^2^ values ​​presented small to moderate effects, ranging from 1.77 to 16.8%.

**Table 3:** Map, linkage group (LG), flanking markers, position of the QTL in centiMorgans (cM position), LOD score, proportion of phenotypic variation (R^2^) explained by QTL in % and additive effect by the QTLs mapping

Map	LG	Flanking markers	cM position	LOD score	R^2^ (%)	Additive effect
*P. trifoliata*	3	100,025,033|F|0	109.81	5.14	1.77	−0.03
*P. trifoliata*	5	100,029,470|F|0	58.51	4.06	6.3	−0.05
*P. trifoliata*	7	100,087,520|F|0	190.66	4.25	7.41	0.05
*C. sunki*	2	100,088,096|F|0	61.74	7.67	16.8	−0.08

### eQTLs analysis and mapping

Using the two genetic maps previously constructed for the progeny of the citrandarins [22] and according to gene expression profile from relative expression values (log_2_Fold change) of 19 target genes evaluated with the 51 selected hybrids, it was possible to detect eQTLs in response to infection caused by *P. parasitica*. Only those with LOD score higher than or equal to 3.0, calculated by the random permutation test for each analyzed gene, with a confidence interval of 95%, were considered as consistent eQTLs. In this model, there is only one effect’s type (additive effect) and the signal indicates the linkage phase between the marker and the eQTL.

**Fig. 7:**
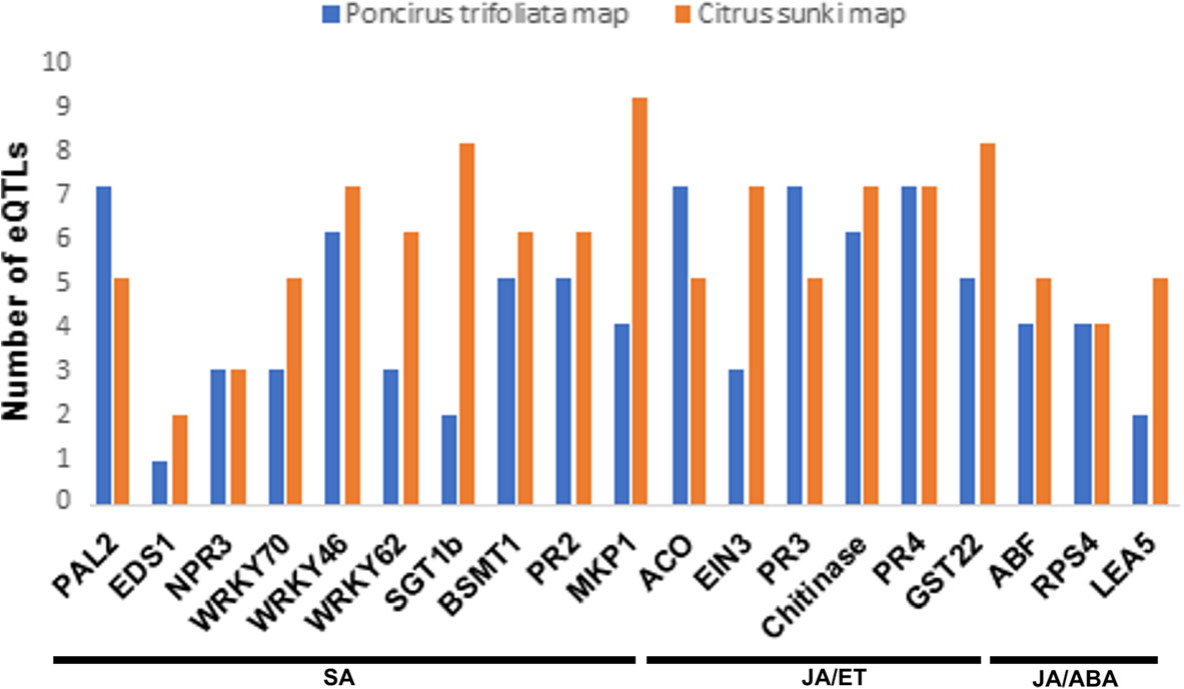
Distribution of the number of eQTLs detected for each candidate gene in relation to the *Poncirus trifoliata* and *Citrus sunki* maps

**Fig. 8:**
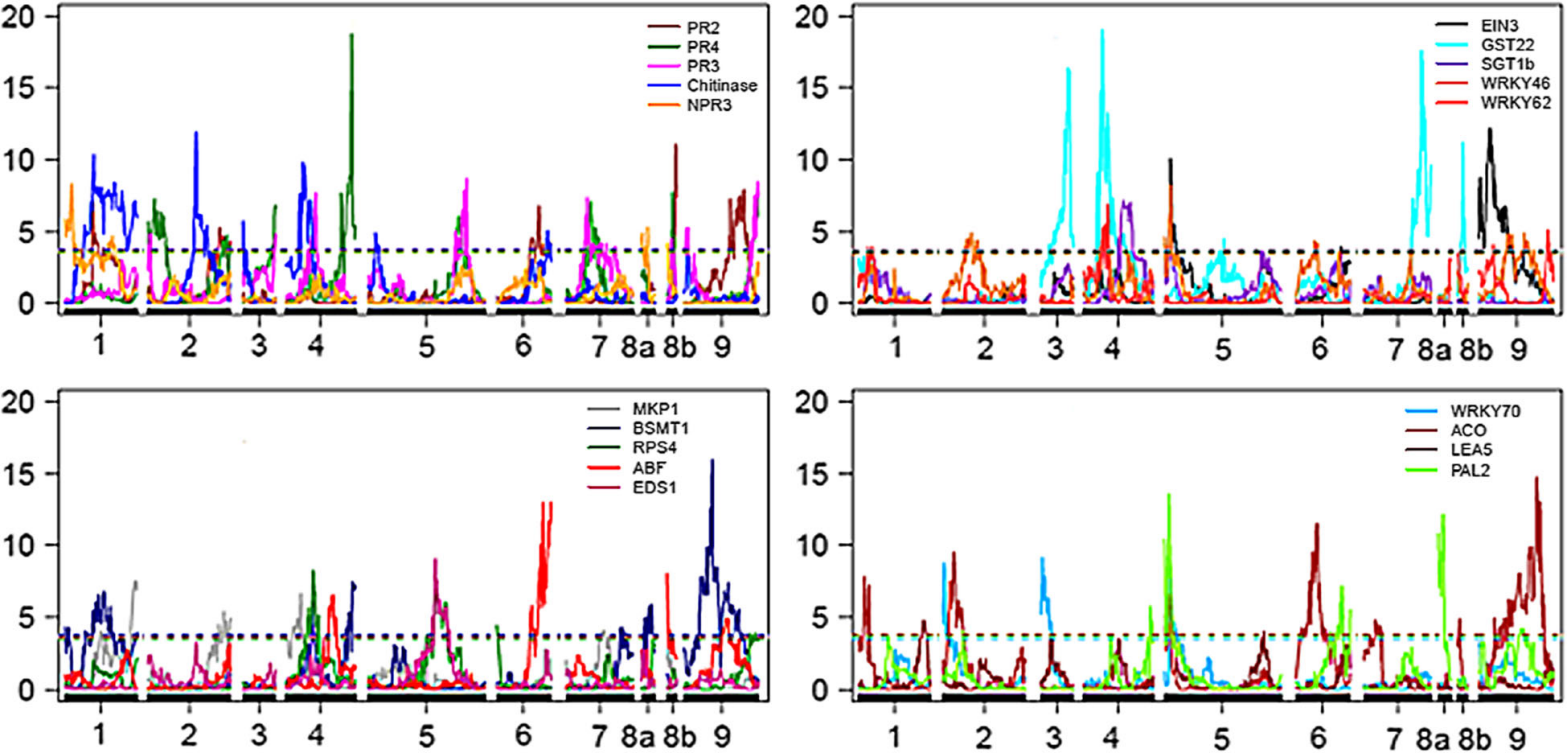
Detection of eQTLs in the *Poncirus trifoliata* map related to expression of 19 target genes. Y-axis: LOD; X-axis: distance in centiMorgans; LOD score = 3.0

**Fig. 9:**
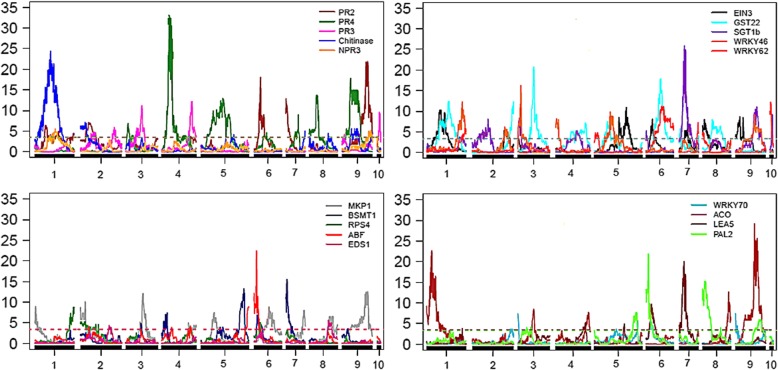
Detection of eQTLs in the *Citrus sunki* map related to expression of 19 target genes. Y-axis: LOD; X-axis: distance in centiMorgans; LOD score = 3.0

According to the methodology of composite interval mapping (CIM), 164 eQTLs with LOD ≥ 3.0 were detected for 19 evaluated genes: 84 of them for the *P. trifoliata* map, with mean of approximately four eQTLs per gene and 1:1 segregation due to the significant additive effect for this parent; and 110 for the *C. sunki* map, with mean of approximately six eQTLs per gene that showed 1:1 segregation, with significant additive effect for this parent. All target genes possessed at least one eQTL associated with their expression on both the Rubidoux trifoliate map and the Sunki mandarin map (Figs. 7, 8 and 9).

In general, the calculated LOD scores were close to 3.0 for all evaluated genes in the two maps. The LOD scores of the eQTLs were consistent and ranged from 3.58 to 19.02 for the *P. trifoliata* map (Additional file 4: Table S4), while ranging from 3.39 to 33.13 for the *C. sunki* map (Additional file 5: Table S5).

Twenty three hotspots were identified on the Rubidoux trifoliate map, with mean of approximately two hotspots per LG, as well as 31 hotspots on the Sunki mandarin map, with approximately three hotspots per LG. Genes that had overlapping eQTLs localized in hotspots ranged from two to eight on genetic maps (Table 4). In addition, the interposition between an eQTL from the *WRKY46* gene and a QTL in the same genomic position was found exactly on the marker 100,087,520|F|0 in the LG7 of the *P. trifoliata* map at 190.66 cM, indicating a complex network that regulates both gene expression and phenotype.

**Table 4:** Relationship of genes that presented overlapping eQTLs located in hotspots for each linkage group (LG) in the two genetic maps built for each parent of the F1 progeny of citrandarins

*Poncirus trifoliata* map		*Citrus sunki* map	
LG	Genes	LG	Genes
1	PR2, Chitinase and PAL2 NPR3, WRKY62 and ACO MKP1 and LEA5	1	PR4, SGT1b, WRKY46 and WRKY70 PR3-Chitinase-GST22-ACO^a^, MKP1, BSMT1-ABF^a^ and PAL2
		2	PR2 and ABF
			PR3, EIN3 and WRKY46
			Chitinase-MKP1-RPS4^a^ GST22 and WRKY70
2	PR4 and PR3 Chitinase, EIN3-SGT1b-WRKY46-WRKY70^a^, ACO-PAL2^a^ and LEA5 RPS4-EDS1^a^ and GST22		
		3	Chitinase, EIN3 and LEA5
			MKP1-GST22^a^ BSMT1, WRKY70 and PAL2
3	PR2 and MKP1 PR4, PR3 and WRKY70		
		4	PR2 and BSMT1-GST22^a^
			Chitinase, MKP1 and WRKY62 SGT1b, WRKY46 and LEA5
4	PR2 e ACO PR4-PR3^a^, RPS4 and WRKY62 ABF, WRKY46 and PAL2		
5	PR2, EIN3 and PAL2	5	PR2-MKP1^a^ and PAL2
	Chitinase and ABF		PR4 and BSMT1 PR3 and EIN3 SGT1b and WRKY46-ACO^a^
	WRKY46 and ACO		
6	NPR3, BSMT1 and PAL2		
7	PR4 and PR3 BSMT1 and GST22^a^		
		6	PR2 and EIN3
			NPR3 and GST22 RPS4 and WRKY62 SGT1b, WRKY46-LEA5^a^ and ACO
8a	PR4 and BSMT1-PAL2^a^		
	PR3, RPS4 and WRKY62		
	Chitinase, MKP1 and GST22		
		7	PR2-RPS4-EIN3^a^
			PR4, MKP1 and GST22 BSMT1, ABF-PAL2^a^ and SGT1b WRKY70 and LEA5
8b	PR2, PR4-GST22^a^, PR3, NPR3, ABF and ACO		
9	PR4, PR3 and GST22 Chitinase and ACO	8	PR4 and GST22
			Chitinase-EDS1^a^
			MKP1 and ACO
			EIN3, WRKY62 and PAL2
		9	PR2, MKP1, RPS4, ABF and GST22-LEA5^a^ PR4 and BSMT1
		10	PR3, NPR3, SGT1b and WRKY62

^a^Genes that presented located eQTLs exactly in the same position in centiMorgans

## Discussion

The RLL means of the hybrids ranged from 1.15 to 11.13 mm, which is in accordance with previous study carried out by Siviero et al. [2], that worked with the same population, using as inoculum a 3 mm disc (cork borer) of oomycete mycelium. The parents represented the extreme points in lesion lengths, with about 15.10 mm for *C. sunki*, and 2.30 mm for *P. trifoliata*. The lesion length values for the F1 individuals ranged from 5.8 to 12.2 mm (discounting 3 mm from the mean values obtained) [2]. In our study, the parents had very contrasting results for the lesion developed by the infection with *P. parasitica*, with *P. trifoliata* (Rubidoux trifoliate) reaching 2.71 mm and *C. sunki* (Sunki mandarin), 8.36 mm (Fig. 1). According to the literature, Sunki mandarin is susceptible to *Phytophthora*, while trifoliate behaves as resistant rootstock [29, 30]. The lesion in the Rangpur lime reached 10.52 mm, surpassing the Sunki mandarin, in order to be classified as susceptible; and in the Swingle citrumelo, the RLL mean was 1.72 mm, reaching values smaller than *P. trifoliata*. Swingle citrumelo rootstock is known as being tolerant for trunk and root infections [31].

The results also indicated that hybrids from the crosses between Sunki mandarin and Rubidoux trifoliate present great genetic variability, being few (17%) susceptible or moderately susceptible to *Phytophthora* (Additional file 3: Table S3).

The value of broad-sense heritability coefficient in Table 1 indicates that 65% of the phenotypic variation is due to the action of genes, demonstrating good genetic control and great chance of success in the selection of individuals within the experiment. This result disagrees with Siviero [32], which found a low value of heritability (18.7%) when evaluating the lesion caused by inoculation of *P. parasitica*.

Considering gene expression results, the M values of endogenous normalizing genes are in accordance with the literature [33] and with the GeNorm manual, because they are below the limit of 1.5 (Table 2). Thus, *FBOX* and *SAND* genes were classified as the most uniform by both the SD and the GeNorm algorithm, surpassing the *GAPC2* housekeeping gene, which is commonly used for the normalization of gene expression assays by qPCR in plants [34, 35].

Three expression patterns were found after the F-test and paired Student’s t-test (Fig. 2). In the first one, there was a significant difference within the MS and T/R groups for the *Chitinase* gene (Fig. 2a). The expression of *Chitinase* was down-regulated in these two groups, but there was a higher repression in the MS group. According to Punja & Zhang [36], there are different classes of plant chitinases from molecular, biochemical and physicochemical criteria, that are constitutively expressed at high levels in the plant in response to several abiotic and biotic factors. In addition, the same author stated that the level of protection is variable in transgenic plants that express cloned genes of chitinases in response to a fungal pathogen.

In the second pattern, significant differences for the *WRKY62* gene were found when comparing log_2_Fold change means between S and MS, as well as MS and T/R (Fig. 2b). In relation to the combination S and MS, this gene was found induced in most genotypes belonging to the MS group, predominantly evidencing the existence of S hybrids that repressed the *WRKY62* gene. This may show a small participation of this gene in the defense response to *P. parasitica*, considering that genotypes with intermediate lesion lengths express it more than others with larger lesion length. On the other hand, the results with the combination MS and T/R indicate that tolerant and/or resistant hybrids of *C. sunki* x *P. trifoliata* don’t response to *P. parasitica* infection with WRKY62 gene, since the mean is down-regulated for this group and consequently smaller than the mean of the MS group, which was up-regulated. Previous studies state that the transcription factor *WRKY62* has a different performance in comparison to other members of the WRKY family, negatively regulating the SA-mediated response and positively regulating a typical PTI (PAMP-triggered immunity) response, by interacting with an also negative regulator of the SA downstream pathway called *HDA19* (histone deacetylase 19) [37, 38].

In addition to the second pattern of expression, a third one was detected, in which there were significant differences for three genes (*EDS1*, *WRKY46* and *LEA5*) when comparing the log_2_Fold change means between S and MS, as well as T/R and S (Figs. 2c, d and e). The genes were induced in most genotypes belonging to the S group, evidencing the predominant existence of MS and T/R hybrids that repressed the *EDS1*, *WRKY46* and *LEA5* genes.

Perhaps resistant citrandarins activate other defense mechanisms, such as the production of ROS (Reactive Oxygen Species) and phytoalexins that are enough to contain the advance of *P. parasitica*. In the literature, it is described that resistance to pathogenic fungi belonging to the genus *Verticillium*, which attack the tomato crop, involves a more basal and nonspecific response, with resistant plants producing depositions (callose, among others) on cell walls and synthesizing antimicrobial agents, including phytoalexins [39]. In addition, previous studies using the microarray technique and qPCR to evaluate differential gene expression in susceptible and resistant tomato varieties to the isolates of *Verticillium dahliae*, identified genes induced to this pathosystem, such as those coding for PR proteins; and found a larger induction, as well as a larger number of genes differentially expressed in susceptible plants to the pathogen. Thus, these results indicate that the increase of systemic gene expression in susceptible plants does not protect them, and that this exaggerated and inductive reaction may trigger the symptoms of the disease [40–42]. In fact, according to Dalio et al. [43], in the susceptible plant (*C. sunki*), *P. parasitica* deploys effectors, such as elicitins, NPP1 (Necrosis-inducing *Phytophthora* protein 1), CBEL (Cellulose-binding elicitor and lectin activity), RxLR and CRN (Crinkler), whereas the host responds by activating the main defense signaling pathways, resulting in hypersensitive response and necrosis. Despite its strength, this defense response fails to withstand *P. parasitica* invasion, confirming its hemibiotrophic life style. In *P. trifoliata*, the effectors were strongly expressed, nevertheless failing to induce any immunity manipulation and disease development, suggesting a non-host resistance type, in which the plant relies on preformed biochemical and anatomical barriers. The *LEA5* gene, in addition to play an important role in resistance to abiotic stresses, may also be related in defense responses to pathogen infection, being responsive to increased levels of JA and ABA [44]. The transcription factor *WRKY46*, as well as *WRKY70*, acts as a positive regulator of the SA signaling pathway [45]. At last, the *EDS1* gene acts on the SA upstream pathway, and consequently in its biosynthesis from an ETI (effector-triggered immunity) [46, 47].

In the cluster analysis of the first heatmap (Fig. 3), there was a higher variation in log_2_Fold change values ​​within the sampled individuals than in the genes. The *BSMT1* and *SGT1b* genes were predominantly up regulated. These genes are part of the SA metabolism [47] and may therefore have a greater participation in the resistance of this population to *P. parasitica*. On the other hand, most hybrids and four analyzed rootstocks were down-regulated for the *EIN3* gene (Fig. 3), an important regulator of the ET downstream pathway [48], indicating that it may not interfere in the defense response of the different phenotypes to *P. parasitica*.

In relation to the genotypes, it was found that the Sunki mandarin and Rangpur lime rootstocks, described in the literature as susceptible to *Phytophthora*, were close (same cluster), showing a similar gene expression profile. A similar event occurred for the Rubidoux trifoliate and the Swingle citrumelo, which are resistant to the oomycete. In addition, hybrids 43, 203, 225 and 285, belonging to the T/R group and to the same cluster in the heatmap, had the highest expression of the 19 evaluated target genes, strongly inducing genes like *BSMT1* and *ABF*. Thus, they are promising materials for understanding the resistance to *P. parasitica* and can be selected for future research related to citrus gummosis disease (Fig. 3). On the other hand, tolerant and/or resistant hybrids, such as 270, 283 and 306 (the last two are in the same cluster) predominantly repressed the candidate genes, suggesting that they probably develop other defense mechanisms against infection caused by *P. parasitica*. For hybrids belonging to the S and MS groups, inferences can also be made for the genotypes 11 and 99, for example, since they presented most of the genes down-regulated, evidencing that they have low resistance to *P. parasitica*. At the same time, many susceptible hybrids (8, 204, 209, 40, 13, 27 and 284) sometimes suppressed some genes, or induced other genes, being grouped in different clusters, what contributes to the idea that different defense mechanisms associated with plant resistance can be established against infestation of *P. parasitica*.

The comparison of the congruence pattern of the gene expression is presented in Fig. 4. The Euclidean Distance is widely used as a parameter to investigate how much an object differs from the other, being efficient to evaluate the intravariable divergence [49]. The darker the color, the more intense the interaction between two genes, demonstrating a larger congruence between expressions. Therefore, smaller distance was observed between the *PR2* and *EDS1* genes, which are part of SA signaling pathway. *EDS1* acts on the biosynthesis of this phytohormone and *PR2* consists of a responsive defense gene to SA accumulation. *PR2* encodes the β-1,3-glucanase protein with anti-oomycetes properties, because it constitutes an enzyme that degrades the cell wall of these organisms that is composed of β-glucans [37]. However, the less intense the color, the more distant the genes will be, which denotes a smaller congruence of the expressions between genes. It was found that three genes (*BSMT1*, *PR4* and *ABF*) in comparison with *WRKY46* obtained the largest distances. It is known that *WRKY46* is a transcription factor belonging to the large WRKY family, and acts as a positive regulator of the SA downstream pathway. The *BSMT1* gene is known to be part of SA metabolism, converting this phytohormone into MeSA (Methyl Salicylate), and has functions as a defense signal for the expression of JA-dependent defense genes. The *PR4* gene encodes a chitinase (PR protein) that is activated by the JA pathway in the ERF branch, contributing to the synergy between the JA/ET pathways. The *ABF* gene is essential in downstream regulation of the ABA pathway, inducing the expression of many responsive defense genes to this gaseous hormone [45, 50, 51]. Hence, there is a smaller relationship between their expressions when evaluating the specific resistance response to *Phytophthora*.

Regarding the QTL mapping in both linkage maps (Figs. 5 and 6), Siviero et al. [2] in their study of QTLs mapping for evaluation of resistance to *Phytophthora* in hybrids of *C. sunki* x *P. trifoliata*, using maps previously constructed by Cristofani-Yaly et al. [52], also found three QTLs for the Rubidoux trifoliate parent. In addition, Siviero et al. [2] also detected only one QTL for Sunki mandarin.

According to Anderson et al. [53], many studies with mapping reveal QTLs in a few loci with moderate R^2^ values (10 to 20%), while there are several other loci with small effects (R^2^ smaller than 10%), which is similar to those found in the present study. QTLs identified in LGs 3, 5 and 7 on the *P. trifoliata* map explain 1.77; 6.3 and 7.41% of the phenotypic variation, respectively. The QTL for *C. sunki* parent, located in LG 2, was responsible for most of the phenotypic variation of the RLL values, explaining 16.8% of it (Table 3). Thus, this suggests that Sunki mandarin has greater contribution in transmitting alleles for gummosis response to the progeny. Some studies with QTLs mapping in other plants reported about resistance alleles located in the susceptible parent, as well as susceptibility alleles in the resistant one [54, 55]. In addition, Siviero et al. [2] also detected a QTL with moderate effect, which explained 18% of the phenotypic variation.

The high value found for heritability (65%) shows that the genetic nature presents a predominant role in the phenotype manifestation, a fact that was not shown in previous studies, in which heritability values ​​found for the character were low, reaching less than 20%, as in the study of Siviero et al. [2].

The detection of several QTLs associated with resistance to *Phytophthora* and the variation of RLL phenotypic data ​​of the hybrids in response to inoculation with *P. parasitica* are indicative that the lesion length character is complex, being controlled by polygenes, as mentioned in the literature [30, 31]. This study of *Phytophthora*-citrus pathosystem demonstrates a great possibility of success in the selection of resistant hybrids within the experiment, an important scenario in a breeding program. The QTLs location and the understanding of their genetic effects contribute to the detection and selection of possible candidate genes responsible for the variation of the studied phenotype [56].

According to Shi et al. [57], a high detection power of eQTLs can be found even in small populations (population size below or close to 50). In addition, as the methodology established by the software for QTLs mapping is similar to eQTLs mapping, a population size larger than 50 is sufficient to provide high resolution to the activity of mapping and detecting QTLs/eQTLs of small effect. The larger the population size, the saturation of the genetic map and the heritability of the phenotypic character, the larger the genomic information related to the mapping [58–60]. As a high heritability (65%) for the RLL character was found in the present study, a high saturation of the two maps of each parent and the population of selected hybrids for the eQTLs mapping being equal to 51, a large volume of eQTLs from the differential gene expression profiles for each candidate gene analyzed via RT-qPCR was found.

*PR4*, *PR3*, *ACO* and *PAL2* genes presented the highest number of eQTLs on the *P. trifoliata* map, each one with six of them, while on the *C. sunki* map there was a larger number for the *MKP1* gene, totalizing nine eQTLs (Fig. 7). The *PR4*, *Chitinase* and *PR3* genes, which are responsive to the JA/ET pathways, encode a class of enzymes called chitinases, which degrade cell wall [61]. They detained a larger number of chromosomal regions that affect their transcription levels in response to infection caused by *P. parasitica* on both the Rubidoux trifoliate map and the Sunki mandarin map. On the other hand, a smaller number (only one eQTL) for the *EDS1* gene was found, which is below the mean of eQTLs per gene in both maps. One of the main regulators of the SA upstream pathway, *EDS1* acts to promote SA biosynthesis due to effector-triggered immunity (ETI) [47]. This gene may not have much participation in the defense response of the interaction *Phytophthora*-citrus, a fact explained by its expression pattern and by smaller quantity of genomic regions in the two contrasting parents, responsible for the variation of its gene expression in the segregating progeny.

Regarding the phenotypic variation (R^2^) values ​​explained by the eQTLs, both on the maps of the Rubidoux trifoliate and the Sunki mandarin (Additional file 4: Table S4 and Additional file 5: Table S5), R^2^ values ​​​​reached up to 11.54% for the *P. trifoliata* map; and up to 24.97% for the *C. sunki* map. Equivalent to the phenotypic variation values ​​explained by QTLs in mapping studies [53], many eQTLs in the present study are composed of loci with small effects (R^2^ less than 10%), while few are formed by chromosome regions with moderate effects (10 to 20%).

Studies with eQTLs allow the identification of chromosomal regions that affect the expression of multiple genes (hotspots). Such phenomenon can be seen by a series of overlapping eQTLs on the LGs of genetic maps, and its consequence can be attributed to genome regions rich in closely linked genes, by identifying nearby eQTLs; or to a single genomic region regulating the transcription levels of many genes, from the detection of distant eQTLs. Nonetheless, it is reported in the literature that many hotspots control the expression of enzyme-coding genes involved in various metabolic pathways, and some hotspots may even regulate the pathway as a whole [62–65].

It could be verified that there was an intense overlap of eQTLs from the quantification of the 19 gene transcripts (Table 4), independent of the signaling pathway involved, as observed in Figs. 8 and 9. Thus, the interposition between eQTLs does not seem to follow the relationship between the genes, including eQTLs from the transcripts associated with different signaling pathways of phytohormone, which were overlapping exactly at the same position or in the proximal regions of hotspots, both on the *P. trifoliata* map and on the *C. sunk* map.

Co-regulated genes in plants have been extensively identified in genetic analyses by eQTLs mapping, with the concept that different transcription levels from distinct genes may be under control of the same loci regulators. In addition, this type of methodology allows the detection of other regulatory networks, from the co-location between QTLs and eQTLs, identifying associations between the allelic state of a genomic region with the quantification of gene transcripts within QTLs involved in the variation of complex agronomic traits. The overlap of QTLs and eQTLs may corroborate to a strong association between gene transcript levels and phenotypic data [66–68]. The *WRKY46* gene is a transcription factor of the WRKY family and its activation is involved in plant responses against phytopathogen infection [37, 45]. It has a gene expression pattern and co-location of QTL and eQTL in *P. trifoliata* map that suggest a larger participation in the interaction of *P. parasitica* with hybrids of *C. sunki* x *P. trifoliata*.

Kirst et al. [69], using the pseudo-testcross strategy to map eQTLs on two parental genetic maps in hybrids of eucalyptus, suggested that different genotypes of the progeny have distinct regulatory loci in the parents, which may explain the genetic differences in the gene expression. In the present study, the susceptible parent Sunki mandarin seemed to have a greater contribution in the transmission of alleles to the progeny, since it was detected in comparison with the map of the trifoliate Rubidoux: a single QTL of moderate effect; higher number of eQTLs; and greater number of hotspots and consequently greater overlap. However, it is worth mentioning that there was an expressive identification of QTLs, eQTLs and hotspots in the Rubidoux trifoliate map, suggesting that there is an interaction between the two parents to confer a favorable combination of QTLs/eQTLs to be transmitted to citrandarins in response to infection of the causal agent of citrus gummosis disease.

According to Dalio et al. [43], that studied the contrasting interactions of Sunki mandarin (susceptible) and trifoliata Rubidoux (resistant) in *Phytophthora*, they noticed that SA, JA and ET pathways seem to be activated in susceptible rootstock upon infection. The plant responds by strongly activating several genes related to all three main defense signaling pathways (SA, JA and ET). This major transcriptional shift results in HR. Nonetheless, this is not efficient to kill or stop pathogen colonization. Since *P. parasitica* has a hemibiotrophic life style, it might benefit from cell death and release of nutrients. On the other hand, trifoliata Rubidoux, even in contact with several *P. parasitica* effectors, does not activate SA, JA or ET pathways.

In our work, in relation to inoculation, the bark was removed as according to Boava et al. [11]. These authors reported the induction of defense-related genes in *P. trifoliata* plants and smaller lesion induced by the pathogen, when compared to *C. sunki*. We also observe this fact, but we observed in general that the eQTLs for *C. sunki* had higher values of phenotypic variations (R^2^) than those observed for *P. trifoliata*.

As argued by Dalio et al. [43], inoculation method by wounding used in our work might favor a first establishment of the pathogen and a concomitant defense response of the plant in *P. trifoliata*, which impairs further development of the pathogen. But we also observed defense response in Sunki and it was not enough to contain the infection. So it seems to us that the genes studied here are not the main ones involved in resistance. It appears that *P. trifoliata* and citrandarins presents other defense mechanisms that may be related to structural and chemical barriers as well as hypothesized by Dalio et al. [43].

## Conclusions

We have identified citrandarins that are promising genotypes for future research related to gummosis disease. The heritability for the lesion length character caused by *P. parasitica* is considered high, demonstrating that genetics exerts larger influence than the environment. From our genetic maps constructed for both parents of the segregating progeny, QTLs that act in the process of expression of the lesion length character related to the inheritance of the citrus gummosis have been identified. The performance of both parents is of extreme importance for the transmission of a favorable combination of QTLs to their hybrids.

This is the first study of functional genomics involving eQTLs mapping in the *Phytophthora*-citrus interaction. Genomic regions in the maps of both parents that act on the gene expression process for the lesion length character associated with citrus gummosis disease were mapped, identifying a series of overlapping eQTLs and consistent hotspots related to the 19 target genes. In addition, our results indicated the formation of a complex regulatory network of both gene expression and phenotype, by the co-location of a QTL with an eQTL in the same marker on the *P. trifoliata* map. As for our QTL mapping, the eQTLs mapping revealed that the susceptible parent Sunki mandarin seemed to have a greater contribution in transmitting alleles to the progeny. The mutual action of both parents is indispensable for the transmission of a favorable combination of eQTLs to citrandarins, which confer defense against *P. parasitica*.

Future prospects can be made as to the exact location of the 19 target genes among the genetic markers distributed on both parental maps, which allows the distinction of the various eQTLs detected in the present study. These can be categorized as cis-eQTLs (eQTLs close to the gene), explaining the variation of gene expression in the chromosomal region in which the gene is found; or trans-eQTLs (eQTLs distant from the gene), representing an effect of genetic polymorphisms that are located in other regions of the genome. This procedure brings larger genomic information to eQTLs mapping, and may contribute to advances in the area, since studies with eQTLs using the qPCR technique in plants are scarce in the literature.

## Additional files

Additional file 1: Selected genes for RT-qPCR assays. (XLSX 11 kb)

Additional file 2: Gene expression data with mean Ct and SD for 19 candidate genes. (XLS 2436 kb)

Additional file 3: Real lesion length (RLL) caused from the inoculation with *P. parasitica* in the individuals of the experiment. (XLSX 12 kb)

Additional file 4: Gene, linkage group (LG), flanking markers, position of the eQTL in centiMorgans (cM position), LOD score, proportion of phenotypic variation (R^2^) explained by eQTL in % and additive effect by the eQTLs mapping in the *Poncirus trifoliata* map. (XLSX 16 kb)

Additional file 5: Gene, linkage group (LG), flanking markers, position of the eQTL in centiMorgans (cM position), LOD score, proportion of phenotypic variation (R^2^) explained by eQTL in % and additive effect by the eQTLs mapping in the *Citrus sunki* map. (XLSX 18 kb)

## Abbreviations

ABA: Abscisic acid
CitEST: Citrus Expressed Sequence Tags
eQTL: Expression Quantitative Trait Loci
ET: Ethylene
HR: Hypersensitivity reaction
JA: Jasmonic acid
JA/ABA: Jasmonic acid and/or Abscisic acid
JA/ET: Jasmonic acid and/or Ethylene
LG: linkage group
LLC: Lesion length in control
LLI: Lesion length by inoculation
MS: Moderately susceptible
QTL: Quantitative Trait Loci
RLL: Real lesion length
RNA-Seq: RNA Sequencing
ROS: Reactive Oxygen Species
RT-qPCR: Real Time quantitative PCR
S: Susceptible
S/MS: Susceptible and/or Moderately susceptible
SA: Salicylic acid
T/R: Tolerant and/or Resistant
